# PHILOS plating versus percutaneous K-wire fixation in proximal humerus fracture management

**DOI:** 10.6026/973206300222900

**Published:** 2026-05-31

**Authors:** Moniruzzaman M.D, Krishna Priya Das, Saraf Anjum, Murad Ahmed S.K, Jiaul Islam M.D, Rafiqul Islam M.D, Bipul Kumar Das

**Affiliations:** 1 Department of Orthopaedic Surgery, National Institute of Traumatology and Orthopaedic Rehabilitation (NITOR), Dhaka, Bangladesh; 2 Department of Orthopaedic Surgery, Bangladesh Medical University, Dhaka, Bangladesh; 3 Department of Obstetrics and Gynaecology, Dhaka Medical College, Dhaka, Bangladesh; 4 Department of Hand & Microsurgery, National Institute of Traumatology and Orthopaedic Rehabilitation (NITOR), Dhaka, Bangladesh

**Keywords:** Proximal humerus fracture, proximal humeral internal locking system (PHILOS) plating, K-wire fixation

## Abstract

Functional outcomes of PHILOS plating versus percutaneous K-wire fixation for managing proximal humerus fractures (PHFs) are debatable since years. Hence, a prospective observational study of 42 patients revealed that PHILOS plating resulted in significantly earlier radiological union and superior shoulder function scores, though with longer operative times and greater blood loss. K-wire fixation was associated with a shorter surgical duration and minimal blood loss with a higher incidence of complications like malunion and pin tract issues. Thus, we show that PHILOS plating offers a more stable fixation for optimized functional recovery, while K-wire fixation remains a viable, less invasive option for selected cases.

## Background:

Proximal humerus fracture (PHF) is one of the most frequently encountered fractures. It represents around 4-5% of all skeletal fractures. It ranks as the third most common fracture in the elderly population, after hip and distal radius fractures [1]. Its incidence has been steadily increasing. This is primarily due to the growing proportion of elderly individuals with osteoporosis. The higher risk of falls in this population also contributes [2]. The bimodal distribution of PHFs is a characteristic feature. Younger adults typically sustain such injuries from high-energy trauma. Examples include road traffic accidents or sports. Older patients are more likely to experience them following low-energy falls, often from standing height [2. This diversity in etiology creates challenges in management. Both patient profile and fracture pattern influence treatment decisions. The management of PHFs depends on several factors. These include fracture type, degree of displacement, bone quality, the patient's age and activity level. Non-displaced and minimally displaced fractures can often be treated conservatively. This approach yields satisfactory results. However, surgical intervention is usually warranted for displaced, unstable, or complex fractures. Surgery aims to restore the anatomy. It also minimizes disruption to the vascular supply. Furthermore, it promotes early mobilization of the shoulder [3]. Early mobilization is especially important to avoid stiffness and functional impairment. Over the years, multiple surgical techniques have been developed to address PHFs. These include intramedullary nailing, tension band wiring, hemiarthroplasty and plate osteosynthesis. However, two fixation methods have emerged as widely practiced in recent decades. These are the Proximal Humeral Internal Locking System (PHILOS) plating and percutaneous Kirschner (K)-wire fixation [4]. These two methods represent fundamentally different philosophies of fracture management. PHILOS plating is a form of open reduction and internal fixation (ORIF). It is designed to provide angular stability, especially in osteoporotic bone. The locking mechanism between the screw and plate creates a fixed-angle construct. This enhances stability and decreases the risk of screw loosening [5]. Studies have demonstrated that PHILOS fixation allows early mobilization. It also encourages functional recovery. Additionally, it reduces the risk of secondary displacement [6]. However, the technique requires extensive surgical exposure. This may disrupt the vascular supply to the humeral head. It can increase operative blood loss. Furthermore, it may raise the risk of avascular necrosis (AVN) or surgical site infection [7]. Moreover, operative time is typically longer. The procedure also demands technical expertise. Percutaneous K-wire fixation, in contrast, is a minimally invasive method. It is associated with shorter operative duration and minimal blood loss. It also preserves the soft tissue envelope [8]. The technique helps maintain the vascularity of the humeral head. It is relatively simple to perform. Its disadvantages, however, include difficulty in achieving accurate reduction. It offers inadequate stability in osteoporotic bone. There are higher rates of pin tract infection, loosening and malunion. It also has limitations in allowing early physiotherapy [9]. Despite the widespread adoption of both techniques, considerable controversy remains. The debate centers on which fixation method produces superior results. Some investigators have reported better radiological and functional outcomes with PHILOS plating [10]. Others have noted that both methods provide acceptable outcomes. However, they differ in their complication profiles [10, 11]. Therefore, it is of interest to compare surgical, radiological and functional outcomes of PHILOS plating versus percutaneous K-wire fixation for managing proximal humerus fractures (PHFs).

## Methodology and Materials:

This prospective observational study with group comparison was conducted at the Department of Orthopaedic Surgery, Bangladesh Medical University (BMU), Dhaka, Bangladesh and New Life Hospital Limited, Dhaka, Bangladesh. The study duration was from March 2021 to September 2023. A total of 42 patients with proximal humerus fractures were included, with 21 allocated to Group A (PHILOS plating) and 21 to Group B (percutaneous K-wire fixation).

## Selection criteria:

## Inclusion criteria:

[1] Patients aged 18-60 years.

[2] Both genders.

[3] Displaced proximal humerus fractures (≥1 cm displacement and >45°C angulation).

[4] Fracture patterns involving two, three or four parts (Neer's classification).

[5] Duration of injury ≤3 weeks.

## Exclusion criteria:

[1] Displaced fractures.

[2] Open/Pathological fractures.

[3] Patients with other fractures in the same limb.

[4] Extension of shaft fractures into the proximal humerus.

[5] Local soft tissue infection at the surgical site.

[6] Pre-existing neuromuscular disorders or neurovascular injury.

[7] Patients with uncontrolled comorbid conditions.

## Data collection procedure:

After obtaining informed consent, a detailed clinical history and physical examination were recorded. Radiological investigations included plain radiographs and when necessary, computed tomography with 3D reconstruction. Operative details such as duration of surgery, intraoperative blood loss and complications were documented. Postoperative follow-up was conducted at 1, 3, 6 and 12 months. Outcomes were measured using the Visual Analogue Scale (VAS) for pain, Constant-Murley Score for function and the radiological assessment of union.

## Ethical considerations:

Ethical approval was obtained from the Institutional Review Board (IRB) of BMU. Written informed consent was collected from all participants. The confidentiality of patient data was maintained throughout the study and participants were assured that their refusal to participate would not affect their treatment.

## Statistical analysis:

Data were analyzed using SPSS version 26. Quantitative variables were expressed as mean ± standard deviation and compared using Student's t-test or Mann-Whitney U test, as appropriate. Categorical variables were presented as frequencies and percentages and compared using the Chi-square test. A p-value <0.05 was considered statistically significant at a 95% confidence interval.

## Results:

A total of 42 patients with Proximal Humerus Fracture were enrolled on the basis of inclusion and exclusion criteria. They were allotted into 2 groups by odd and even technique, 21 patients in ORIF by PHILOS plate & screws (Group A) and 21 patients in Percutaneous K-wire fixation group (Group B). They were evaluated preoperatively and then followed up at the end of 1 month, 3-month, 6 month and 12 months. Mean age of the study subject was 49.07±9.66, ranging from 27-60 years. 50% of the patients were more than 50 years of age. Male patients were slightly predominant over females, accounting for 52.4% of the cases. Most of the patients were homemakers (33.3%), followed by service holders (31.0%), business (19.0%), unemployed (11.9%) and students (4.8%). 85.7% of the study population was right-handed (Table 1). The right side was involved in 61.9% cases and the left side in 38.1%. Most of the patients had 3-part fractures accounting for 54.7% cases, followed by 2-part fractures (28.6%) and 4-part fractures (16.7%) (Table 2). In Group A, the mean operative duration was 65.52 ±9.32 (60-90) min and in Group B was 36.57± 8.16(28-52) min. The operative duration was significantly longer in Group A than in Group B (p<0.001). In Group A, the perioperative blood loss was 317.14±72.95 (200-500) ml and in Group B, 17.14±9.69 (5-40) ml. The perioperative blood loss was significantly higher in Group A than in Group B (p<0.001) (Table 3). The shoulder forward flexion, abduction, internal rotation in abduction and external rotation in abduction were significantly better in Group A (152.14± 16.70°C, 156.19±18.77°C, 67.14±7.68°C, 73.33±7.80°C; respectively) than in Group B (142.38±16.93°C, 136.43±22.31°C, 59.52±8.93°C, 63.33±7.47°C, respectively) (p=0.009, p=0.001, p=0.002, p=0.002; respectively). In Group A, the radiological union was seen at 9.57±1.89 (6-14) weeks and in Group B were 12.10±2.30 (7-16) weeks. Group A had significantly earlier fracture union than Group B (p=0.001) (Table 4). In Group A, 2(9.5%) cases had surgical site infection and 1(4.8%) had AVN of the head of the humerus. Whereas, in Group B 2(9.5%) had pin tract infection, 2(9.5%) had malunion and 1(4.8%) had pin loosening (Table 5). At 6-month follow up the Constant Murley Score had significantly improved scores in Group A (84.76± 7.04(70-100)) than Group B (77.86± 10.14(58-92)) (p=0.043). In Group A, 42.9% of the cases had excellent outcome, 52.4% had good outcome and 4.8% had moderate outcome; whereas, in Group, B 23.8% of the cases had an excellent outcome, 61.9% had a good and 14.3% had a moderate outcome (Table 6).

## Discussion:

Proximal humerus fractures continue to pose therapeutic challenges to orthopaedic surgeons. This is due to their variable presentation, the influence of bone quality and the need to balance stability with biological preservation. In this study, both PHILOS plating and percutaneous K-wire fixation achieved union in all patients. However, the overall quality of recovery and complication profiles differed significantly between the two methods. The mean age of the study population was 49.07 years. Most patients were in their fifth and sixth decades. This demographic trend is consistent with the findings of Singh et al. and Vijayvargiya *et al.* In their studies, the majority of proximal humerus fractures occurred in middle-aged to older adults [12, 13]. The slight male predominance in this study was also comparable to earlier reports. Chugh *et al.* showed male predominance in their study [1^14^]. Right-sided involvement was more common. Falls on the ground were the leading cause of injury, accounting for 64.3% of cases. These results are similar to those of Ortmaier *et al.* and Daw *et al.* They also reported that simple falls represented the most frequent mechanism in their series [15, 16]. Concerning fracture configuration, three-part fractures were the most common in this cohort. They comprised 54.7% of all cases. When analyzing operative parameters, significant differences were observed between the two groups. PHILOS plating required a mean operative time of 65.52 minutes. This is compared to 36.57 minutes in the K-wire group. Intraoperative blood loss was markedly higher with PHILOS fixation (317.14 ml vs. 17.14 ml). These findings are well supported by Kumar *et al.* and Singh *et al.* They consistently reported that PHILOS fixation demands more surgical time. It is also associated with greater blood loss. This is due to the extensive dissection required for anatomical reduction and plate placement [11, 12]. The minimally invasive nature of percutaneous K-wire fixation explains its shorter duration and lower blood loss. Radiological union occurred significantly earlier in patients treated with PHILOS plating. The mean was 9.57 weeks. This is compared to 12.10 weeks in those treated with K-wire fixation. The earlier consolidation observed with PHILOS may be attributed to the stable fixed-angle construct. This construct allows both maintenance of reduction and micro environmental stability. This stability is conducive to callus formation. K-wire fixation, in contrast, often permits slight motion at the fracture site. This prolongs healing time. Similar findings have been documented by Vijay *et al.* They both observed faster healing in patients treated with locking plates compared to K-wires [17]. Functional outcomes were also superior in the PHILOS group. The mean Constant-Murley Score at six months was 84.76 in the PHILOS group. This is compared to 77.86 in the K-wire group. This difference was statistically significant. A higher proportion of patients in the PHILOS group achieved excellent or good results. The range of motion in all planes was significantly better than in the K-wire group. These planes include flexion, abduction, internal rotation and external rotation. These results are consistent with those reported by Agarwal* et al.* They demonstrated that PHILOS fixation allows earlier mobilization. It also results in improved functional recovery [18]. In terms of pain, although both groups improved substantially over time, PHILOS patients experienced earlier relief. This was particularly evident in the three-month follow-up. Comparable patterns were observed in earlier comparative studies. Complications were encountered in both groups, but their nature differed. In the PHILOS group, there were two cases of surgical site infection (9.5%). There was also one case of avascular necrosis of the humeral head (4.8%). These complications are recognized risks of open reduction and plating. They have been reported by Som *et al.* and Vijayvargiya *et al.* [4, 13]. In the K-wire group, complications included two cases of pin tract infection (9.5%). There were also two cases of malunion (9.5%) and one case of pin loosening (4.8%). These findings are consistent with the observations of Vijan *et al.* They reported pin loosening and infection as the most common problems associated with K-wire fixation [1]. Importantly, no cases of nonunion were observed in either group. The majority of patients expressed satisfaction with their outcome. The overall findings of this study contribute to the growing body of evidence. This evidence supports PHILOS plating as the superior option for displaced proximal humerus fractures. This is particularly true for three- and four-part fractures. It provides earlier union, better functional recovery and improved range of motion compared to K-wire fixation. However, it is also associated with longer surgery. It involves greater intraoperative blood loss. It also carries procedure-specific complications such as surgical site infection and avascular necrosis. K-wire fixation, on the other hand, remains a simpler and less invasive option. It is better suited to patients with comorbidities. It is also suitable for those requiring a shorter operative procedure. This study reinforces the principle that treatment of proximal humerus fractures must be individualized. This requires taking into account fracture configuration, patient age, bone quality and general health. While PHILOS plating may be the preferred option for patients requiring stable fixation and rapid return to function, percutaneous K-wire fixation may still have a role. This role is in selected cases where minimally invasive techniques are advantageous. This is especially true for patients with multiple comorbidities.

## Limitations of the study:

[1] This study was done in tertiary level hospitals, so it doesn't represent the overall scenario of the country.

[2] There was a chance of selection bias due to purposive sampling.

[3] Long term outcome couldn't be assessed due to short follow up duration.

## Conclusion: 

PHILOS plating provides superior radiological and functional outcomes compared with percutaneous K-wire fixation in the management of proximal humerus fractures. Although it is associated with a longer operative duration, higher blood loss and certain complications such as surgical site infection and AVN, the overall patient satisfaction and recovery are significantly better. K-wire fixation, although less invasive and quicker to perform, is limited by higher rates of pin-related complications and poorer functional outcomes.

## Ethical approval:

The study was approved by the Institutional Ethics Committee.

## Figures and Tables

**Table 1:** Demographic characteristics of patients

**Parameter**		**Group A (n= 21)**	**Group B (n= 21)**	**Total (n= 42)**
Age	18-30 years	0 (0)	1 (4.8)	1 (2.4)
	31-40 years	4 (19.0)	4 (19.0)	8 (19.0)
	41-50 years	7(33.3)	5 (23.8)	12 (28.6)
	51-60 years	10 (47.6)	11 (52.4)	21 (50.0)
	Mean ± SD	49.52 ±9.29	48.62 ±10.22	49.07 ±9.66
Gender	Male	12 (57.1)	10 (47.6)	22 (52.4)
	Female	9 (42.9)	11 (52.4)	20 (47.6))
Occupation	Student	0 (0)	2 (9.5)	2 (4.8)
	Home maker	7 (33.3)	7 (33.3)	14 (33.3)
	Business	2 (9.5)	6 (28.6)	8 (19.0)
	Service Holder	9 (42.9)	4 (19.0)	13 (31.0)
	Unemployed	3 (14.3)	2 (9.5)	5 (11.9)
Dominant Hand	Right	19 (90.5)	17(81.0)	36 (85.7)
	Left	2 (9.5)	4 (19.0)	6 (14.3)

**Table 2:** Pre-operative clinical characteristics of patients

**Parameter**		**Group A (n= 21)**	**Group B (n= 21)**	**Total (n= 42)**
Side of Involvement	Right	14 (66.7)	12 (57.1)	26(61.9)
	Left	7 (33.3)	9 (42.9)	16 (38.1)
Mechanism of Injury	Road traffic accident	7 (33.3)	7(33.3)	14 (33.3)
	Fall on ground	14 (66.7)	13 (61.9)	27 (64.3)
	Sports	0 (0)	1 (4.8)	1 (2.4)
Type of injury(Neer's classification)	2 Part	6 (28.6)	6(28.6)	12 (28.6)
	3 Part	12 (57.1)	11 (52.4)	23 (54.7)
	4 Part	3 (14.3)	4 (19.0)	7 (16.7)

**Table 3:** Distribution according to operative duration and blood loss

**Parameter**	**Group A**	**Group B**	**p-value**
Operative duration (min, mean ± SD)	65.52 ± 9.32	36.57 ± 8.16	<0.001
Blood loss (ml, mean ± SD)	317.14 ± 72.95	17.14 ± 9.69	<0.001

**Table 4:** Distribution according to the Range of motion (ROM) and radiological union

**Outcome**		**Group A**	**Group B**	**P value**
ROM of shoulder	Flexion (°C)	152.14 ± 16.70	142.38 ± 16.93	0.009
	Abduction (°C)	156.19 ± 18.77	136.43 ± 22.31	0.001
	Internal Rotation in Abduction (°C)	67.14 ± 7.68	59.52 ± 8.93	0.002
	External Rotation in Abduction (°C)	73.33 ± 7.80	63.33 ± 7.47	0.002
Radiological union	Union Duration (Weeks)	9.57 ± 1.89	12.10 ± 2.30	0.001
	Mean ±SD (Min- Max)	(6-14)	(7-16)	

**Table 5:** Distribution according to postoperative complications

**Complication**	**Group A (n=21)**	**Group B (n=21)**
Surgical site infection	2 (9.5)	0(0)
Pin tract infection	0 (0)	2(9.5)
Pin loosening	0 (0)	1 (4.8)
AVN of head of humerus	1 (4.8)	0 (0)
Malunion	0	2 (9.5)
None	18 (85.7)	16 (76.2)

**Table 6:** Distribution according to post-operative Constant Murley Score

**Constant Murley Score**	**Group A (n=21)**	**Group B (n=21)**	**P value**
Mean ± SD (Min- Max)	84.76 ± 7.04	77.86 ± 10.14	0.043
Excellent (86-100)	9 (42.9)	5 (23.8)	
Good (71-85)	11 (52.4)	13 (61.9)	
Moderate (56-70)	1 (4.8)	3 (14.3)	
Poor (0-55)	0 (0)	0 (0)	
